# Recovery from secondary adrenal insufficiency in a patient with immune checkpoint inhibitor therapy induced hypophysitis

**DOI:** 10.1186/s40425-019-0729-3

**Published:** 2019-09-12

**Authors:** Sahityasri Thapi, Amanda Leiter, Matthew Galsky, Emily J. Gallagher

**Affiliations:** 1 Icahn School of Medicine at Mount Sinai, New York, USA; 2 Division of Endocrinology, Diabetes and Bone Diseases, Department of Medicine, Icahn School of Medicine at Mount Sinai, One Gustave L. Levy Place, Box 1055, New York, NY 10029 USA; 3 Tisch Cancer Institute, Icahn School of Medicine at Mount Sinai, New York, USA; 4 Division of Hematology and Medical Oncology, Icahn School of Medicine at Mount Sinai, New York, USA

**Keywords:** Immune-related adverse events, Immune checkpoint inhibitors, Hypophysitis, Adrenal insufficiency

## Abstract

**Background:**

Hypophysitis is a well-recognized immune-related adverse event in patients treated with immune checkpoint inhibitors for cancer. Some anterior pituitary hormones may recover; however, secondary adrenal insufficiency is usually permanent.

**Case presentation:**

A 26-year old male with metastatic clear cell renal cell carcinoma was started on treatment with the anti-programmed cell death-1 monoclonal antibody (anti-PD-1 mAb) nivolumab, followed by combined nivolumab and the anti-cytotoxic T-lymphocyte-associated protein 4 (CTLA-4) mAb, ipilimumab. After starting nivolumab monotherapy the patient developed thyroiditis, which resolved without treatment. Prior to commencing combined ICI therapy, a random serum cortisol drawn at 1:30 pm and was 15.0 μg/dL (414 nmol/L). Three weeks after starting combined ICI therapy he developed sudden onset of severe fatigue and 1 pm serum cortisol was 2.0 μg/dL (55.2 nmol/L), adrenocorticotropic hormone (ACTH) was 16 pg/mL (3.52 pmol/L). A diagnosis of hypophysitis was made, and he was immediately started on prednisone 1 mg/kg. His symptoms resolved rapidly, and he continued immune checkpoint inhibitor therapy. He was noted to also have low gonadotropic hormones and testosterone (nadir testosterone 81.19 ng/dL). The prednisone was tapered slowly over the next six weeks to a maintenance dose of 5 mg daily. Four months after the initial presentation his cortisol remained low, but his testosterone level had increased to 973.43 ng/dL. After five months his random serum cortisol (1 pm) increased to 11.0 μg/dL (303.6 nmol/L). The prednisone was cautiously discontinued with close monitoring. Two months off glucocorticoid replacement he remained asymptomatic with an ACTH of 24.1 pg/mL (5.3 pmol/L), and cortisol of 13.0 μg/dL (358.8 nmol/L).

**Conclusions:**

This case documents the unusual recovery from secondary adrenal insufficiency in a patient who developed hypophysitis from immune checkpoint inhibitor therapy. Repeated pituitary hormone testing every three months for the first year after the development of hypophysitis may identify more patients with hypothalamic-pituitary-adrenal axis recovery.

## Introduction

Hypophysitis is a well-recognized immune-related complication of immune checkpoint inhibitor cancer therapies [1]. The anti-cytotoxic T-lymphocyte- associated protein 4 (CTLA-4) monoclonal antibody (mAb) ipilimumab is associated with hypophysitis in a dose-dependent manner, with rates up to 21% in patients with melanoma treated with a dose of 9 mg/kg [2]. Hypophysitis is less common with anti-programmed cell death protein-1 (PD-1) and anti-programmed death ligand 1 (PD-L1) mAbs than anti-CTLA-4 mAbs. Combined ICI therapy with the anti-PD-1 mAb nivolumab, and anti-CTLA-4 mAb ipilimumab in clinical trials for melanoma led to higher rates of hypophysitis than with nivolumab monotherapy [3]. Hypophysitis may affect anterior or posterior pituitary function. While the synthesis and secretion of some anterior pituitary hormones may recover, central adrenal insufficiency is usually permanent [4, 5].

The aim of this report is to describe a case of hypophysitis with multiple hormone deficiencies secondary to ipilimumab and nivolumab combined therapy, who recovered all pituitary hormones, including secondary adrenal insufficiency.

## Case description

A 26-year-old male presented to the Cancer Center at Mount Sinai Hospital for evaluation and treatment of metastatic renal cell carcinoma (RCC). He was initially diagnosed with non-clear cell RCC one year earlier in another country, and had a left nephrectomy at that time. After the nephrectomy, he was found to have multiple metastases and was treated with sunitinib, which was discontinued due to a desquamating skin reaction. He then received methotrexate, vinblastine, adriamycin, and cisplatin (MVAC) chemotherapy for five months, and had a partial cancer response. He was subsequently started on sorafenib and gemcitabine, but developed an anaphylactic reaction to sorafenib, and so continued gemcitabine monotherapy. Three months before presenting to Mount Sinai, he had persistent metastatic cancer on whole body fluorodeoxyglucose positron emission tomography computer tomography (FDG PET-CT), and was treated with gemcitabine, cisplatin and paclitaxel. He received intermittent glucocorticoids with chemotherapy but all glucocorticoids were discontinued prior to being seen at our Cancer Center.

**Fig. 1:**
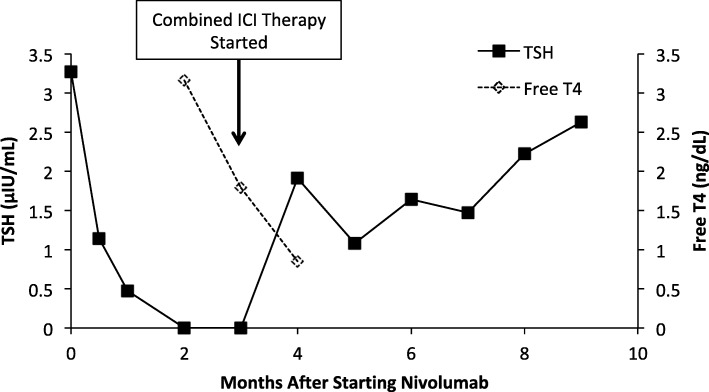
TSH and Free T4 levels after starting nivolumab therapy. Nivolumab started at time 0. Vertical arrow indicates the time at which combined immune checkpoint inhibitor (ICI) therapy with nivolumab and ipilimumab was started. TSH normal range: 0.34–5.6 μIU/mL, free T4 normal range 0.8–1.5 ng/dL

After his initial evaluation at Mount Sinai, he had a CT scan that revealed multiple masses in his adrenals, spleen, and in the peri-aortic region consistent with metastatic disease. His initial tumor pathology specimens were sent to Mount Sinai to be re-examined. The tumor was found to be clear cell RCC (CCRCC) with 50% programmed death ligand 1 (PD-L1) positivity. He was started on nivolumab 240 mg every 2 weeks. He had a normal thyroid stimulating hormone (TSH) of 3.27μIU/mL (normal range [ref]: 0.34–5.6 μIU/mL) prior to starting nivolumab, and developed thyroiditis with hyperthyroidism six weeks later but was asymptomatic (Fig. 1). His anti-thyroglobulin, anti-thyroid peroxidase, and anti-TSH receptor autoantibodies were all negative. Following two months of treatment with nivolumab, CT imaging revealed progression of disease, and he commenced combined immune checkpoint inhibitor therapy with nivolumab (3 mg/kg) and ipilimumab (1 mg/kg). Prior to starting combined therapy, he had normal serum cortisol of 15.0 μg/dL (ref: 6.7–22.6 μg/dL) that was drawn at 1:30 pm.

**Fig. 2:**
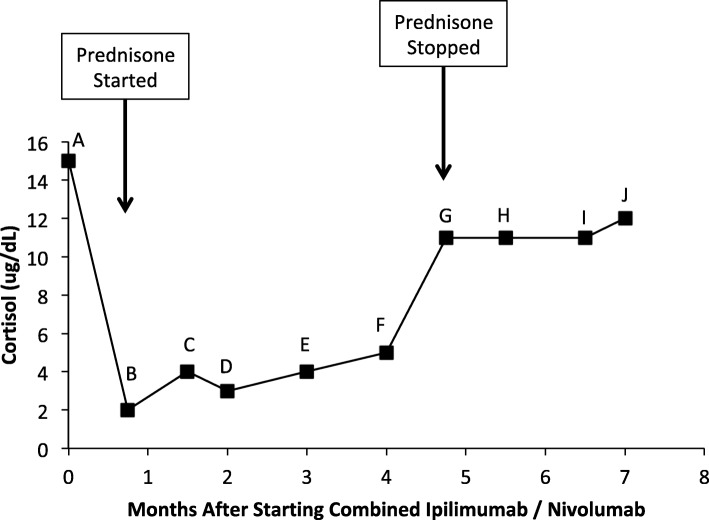
Serum cortisol levels after starting combined immune checkpoint inhibitor therapy. Time 0 is baseline cortisol. Prednisone initiation and discontinuation are indicated by vertical arrows. Serum cortisol values are marked A-J and were drawn at the following times of day – A: 1:30 pm; B: 1 pm; C: 3 pm; D: 2 pm; E: 9 am; F: 1 pm; G: 12:30 pm; H: 12:30 pm; I: 11 am; J: 12 pm. Serum cortisol reference range: 6.7–22.5μg/dL

Three weeks after initiating combined immune checkpoint inhibitor therapy, he complained of sudden onset of severe fatigue, and cold intolerance, but denied headache or visual changes. A random (1 pm) serum cortisol was drawn and was found to be 2.0 μg/dL (Fig. 2), with an ACTH of 16 pg/mL (ref: 7-69pg/mL). Further pituitary hormone evaluation was performed at 4 pm on the same day. His prolactin was elevated at 47.2 ng/mL (ref: 2.6–13.1 ng/mL), total testosterone 545.46 ng/dL (ref: 300-1080 ng/dL), luteinizing hormone (LH) 3.41mIU/mL (ref: 1.2–8.6 mIU/mL), follicle stimulating hormone (FSH) 14.6 mIU/mL (ref: 1.3–19.3mIU/mL), TSH 1.91 μIU/mL, free thyroxine (fT4) 0.85 ng/dL (ref: 0.8–1.5 ng/dL). A diagnosis of hypophysitis was made, and he was immediately started on prednisone 1 mg/kg. A brain magnetic resonance imaging (MRI) reported no pituitary abnormalities. Forty-eight hours after starting prednisone his symptoms resolved. The prednisone was slowly tapered over the next six weeks to a maintenance dose of 5 mg daily. His pituitary hormones were re-assessed one month after the diagnosis of hypophysitis was made. His cortisol at 3 pm was 4.0 μg/dL (Fig. 2), ACTH: 5 pg/mL, total testosterone: 119.45 ng/dL, LH: 3.63mIU/mL, FSH: 18.4 mIU/mL, TSH: 1.62 μIU/mL, fT4: 0.99 ng/dL, insulin-like growth factor (IGF-1): 195 ng/mL (ref: 155-432 ng/mL). He completed four cycles of combined immune checkpoint inhibitor therapy with tumor response on CT. During this time he complained of erectile dysfunction, and his total testosterone was found to reach a nadir of 81.19 ng/dL on a blood test drawn at 2 pm six weeks after the diagnosis of hypophysitis was made.

He followed regularly with endocrinology and remained on prednisone. He was not started on testosterone replacement. His testosterone levels were noted to recover to 973.43 ng/dL on a blood test drawn at 1:30 pm, three and a half months after being diagnosed with hypophysitis. Five months after his diagnosis, his random cortisol at 12:30 pm was found to be 11 μg/dL (Fig. 2), and testosterone was 901.5 ng/dL, with LH: 11.49mIU/mL and FSH: 18.4mIU/mL. He reported missing doses of prednisone, and that if he forgot to take the prednisone he was asymptomatic, while two to three months previously he felt extremely fatigued and unwell if he missed a dose. The prednisone was cautiously discontinued. He remained asymptomatic. Two months after discontinuing the prednisone, his cortisol at 12 pm was 12.0 μg/dL, ACTH 24.1 μg/dL.

## Discussion and conclusions

Hypophysitis is one of the more common endocrine immune-related adverse events observed in patients treated with ICI therapy, particularly anti-CTLA-4 mAb monotherapy, or when combined with anti-PD-1 mAbs.

The mechanisms through which anti-CTLA-4 mAbs cause hypophysitis have been studied in murine models and humans. Hypophysitis has been associated with the development of anti-pituitary autoantibodies, and the direct effects of anti-CTLA-4 mAb on the pituitary [6, 7]. An autopsy examination of a case of hypophysitis induced by anti-CTLA-4 mAb therapy found necrotizing hypophysitis with almost complete destruction of the anterior pituitary [6]. CTLA-4 is expressed on a small number of pituitary endocrine cells, and the level of expression varies between individuals. It has been proposed that anti-CTLA-4 mAb binds to the CTLA-4 expressed on these hormone secreting pituitary cells, leading the formation of immune complexes, subsequent complement activation, and the recruitment of macrophages and other inflammatory cells,resulting in phagocytosis. A lymphocytic infiltration is believed to occur as a later event [6, 7].

Previous case series have reported recovery of thyroid and gonadal axes, but recovery from secondary adrenal insufficiency is extremely rare [8, 9]. Indeed, to our knowledge only two previous cases have been reported [8, 9], and secondary adrenal insufficiency is generally considered permanent [10]. Due to the small number of reports of recovery of secondary adrenal insufficiency, it is currently unknown whether any patient factors (e.g. patient’s age), or treatment strategies (e.g. the rapid initiation of high dose glucocorticoids upon presentation) influence recovery. In one previous case series it was reported that high dose steroids do not alter the outcome of pituitary function recovery [5]. Another possibility is that some patients, such as the patient described in this case, do not have complete destruction of ACTH secreting cells (indicated by low, but detectable plasma ACTH). These patients with low but detectable ACTH may have a greater chance of recovery from secondary adrenal insufficiency. Further case studies may help to understand what factors increase the likelihood of recovery of pituitary function.

The current US prescribing information for ipilimumab recommends holding treatment and initiating glucocorticoids at a dose of 1-2 mg/kg of prednisone or equivalent, in addition to appropriate hormone replacement, in patients who develop hypophysitis (https://packageinserts.bms.com/pi/pi_yervoy.pdf Revised 5/2019, last accessed August 12th 2019). In this case we followed these recommendations and initiated high dose steroids, followed by a rapid taper to physiological replacement doses. In addition to the more commonly considered adverse consequences of glucocorticoid use [11], concerns have been raised as to whether using high doses of steroids to treat irAEs may be associated with reduced tumor response to ICI therapy. A recent retrospective study compared the effect of low-dose and high-dose glucocorticoids on the overall survival (OS) and time to treatment failure (TTF) in patients with melanoma who developed hypophysitis from ICI therapy [12]. Low dose was defined as a maximum average daily dose of 7.5 mg of prednisone or below, and high dose was defined as a maximum average daily dose of greater than 7.5 mg during the initial two month period after the diagnoses of hypophysitis. Both TTF and OS were significantly better in the group that received low dose glucocorticoids, compared with those who received high dose glucocorticoids. Notably, in that study the patients who received high dose glucocorticoids also received significantly fewer total treatment cycles (mean 3.6 ± 0.1), compared with those who were given low dose glucocorticoids (mean 6.4 ± 0.2), which may also have contributed to the observed differences in OS and TTF [12]. Another retrospective study of patients with non-small cell lung cancer treated with anti-PD-1/anti-PD-L1 mAbs, reported a detrimental effect of baseline prednisone use of ≥10 mg/day (or equivalent dosage of another glucocorticoid), compared with < 10 mg/day on overall response rate, progression-free survival, and OS [13]. Not all studies have found that the use of high doses of systemic glucocorticoids for irAEs alter the OS or TTF [14]. Therefore, further studies are needed to examine whether high doses of glucocorticoids adversely affect tumor response and survival, when controlled for differences in treatment duration, and if baseline glucocorticoid use has different effects on tumor response than initiating glucocorticoids to treat irAEs.

Overall, this case documents the unusual recovery from secondary adrenal insufficiency in a patient who developed hypophysitis from combined anti-CTLA-4 and anti-PD-1 therapy. Guidelines suggest retesting of the hypothalamic-pituitary-adrenal (HPA) axis every three to six months in the first year after diagnosis of hypophysitis [10], but it is unknown how frequently re-assessment of pituitary function is performed in clinical practice. As different exogenous glucocorticoids interfere with certain cortisol assays, and long-term glucocorticoid treatment will suppress the HPA axis, it is important that re-evaluation is performed appropriately. Our case report supports the recommendation of frequent retesting of the HPA axis in patients who develop hypophysitis due to ICI therapy. We therefore recommend retesting of the HPA axis every 3 months for the first year after diagnosis of hypophysitis. Careful retesting may identify more patients who recover from secondary adrenal insufficiency, and who may not require lifelong glucocorticoid replacement.

## Data Availability

All data generated or analysed during this study are included in this published article.

## Abbreviations

ACTH: Adrenocorticotropic hormone
CCRCC: Clear cell renal cell carcinoma
CTLA-4: Cytotoxic T-lymphocyte- associated protein 4
FDG PET-CT: Fluorodeoxyglucose positron emission tomography computer tomography
FSH: Follicle stimulating hormone
fT4: Free thyroxine
HPA: Hypothalamic-pituitary-adrenal
ICI: Immune checkpoint inhibitor
IGF-1: Insulin-like growth factor 1
LH: Luteinizing hormone
mAb: Monoclonal antibody
MVAC: Methotrexate, vinblastine, adriamycin, and cisplatin
PDL-1: Programmed death ligand 1
TSH: Thyroid stimulating hormone
